# MicroRNA-223 and microRNA-92a in stool and plasma samples act as complementary biomarkers to increase colorectal cancer detection

**DOI:** 10.18632/oncotarget.7119

**Published:** 2016-02-01

**Authors:** Pi-Yueh Chang, Chia-Chun Chen, Yu-Sun Chang, Wen-Sy Tsai, Jeng-Fu You, Geng-Ping Lin, Ting-Wen Chen, Jinn-Shiun Chen, Err-Cheng Chan

**Affiliations:** ^1^ Department of Laboratory Medicine, Chang Gung Memorial Hospital at Linkou, Taoyuan, Taiwan; ^2^ Department of Medical Biotechnology and Laboratory Science, Chang Gung University, Taoyuan, Taiwan; ^3^ Molecular Medicine Research Center, Chang Gung University, Taoyuan, Taiwan; ^4^ Department of Colorectal Surgery, Chang Gung Memorial Hospital at Linkou, Taoyuan, Taiwan; ^5^ Bioinformatics Core Laboratory, Molecular Medicine Research Center, Chang Gung University, Taoyuan, Taiwan

**Keywords:** microRNA, miR-223, miR-92a, colorectal cancer, stool

## Abstract

Aberrant levels of circulating miRNAs are potential biomarkers for the early detection of colorectal cancer (CRC). However, no previous systematic study has examined miRNAs in various specimen types from the same patient to evaluate their clinical utility. In this study, we compiled information from ∼450 articles published before 2012, and selected the 46 most frequently reported CRC-related miRNAs as candidates. We then established a 46-miRNA multiplex RT-qPCR method, and efficiently examined two clinically accessible samples: stool from fecal occult blood test and EDTA plasma. A total of 62 tissue, 447 stool, and 398 plasma samples were collected from CRC patients and healthy controls. Good correlations of detectable miRNAs were noticed in paired tumor tissues, stool, and plasma samples of 62 CRC patients. Using these 62 CRC patients and 62 matched healthy controls as the training set, 5 and 11 differentially expressed miRNAs achieved the area under the ROC curve (AUC) greater than 0.7 in stool and plasma samples, respectively. The selected miRNAs was subsequently validated using the remaining enrolled samples as the test cohort; 4 miRNAs in stool and 6 miRNAs in plasma were maintained discriminating powers for CRC patients. After examining the complementary effect, combined analysis of miR-223 and miR-92a, which were commonly present in stool and plasma samples, yielded the highest sensitivity of 96.8% and the specificity of 75% for CRC (AUC = 0.907). These results allowed us to establish a two-miRNA biosignature in two types of CRC clinical specimens with a high sensitivity for CRC detection.

## INTRODUCTION

Colorectal cancer (CRC) has a high worldwide prevalence rate and a 5-year survival rate around 60% [1]. CRC arises from the accumulation of genetic alterations in colonic epithelial cells, and the majority of cases follow a tissue sequence of normal-adenoma-carcinoma [2]. The survival rate can increase to 90% when the cancer is detected at an early stage [1]. Numerous lines of evidence support the necessity for CRC screening. Moreover, the removal of adenomatous polyps can reduce their likelihood of progressing to cancer [3]. Colonoscopy is the standard method used to detect these lesions in the intestinal lumen, but population-level compliance tends to be poor because this procedure is invasive and has a risk of perforation. Guidelines also recommend the population-level use of non-invasive stool DNA tests and the fecal occult blood test (FOBT), whose sensitivities vary from 60% to 90% [4]. However, multi-target stool DNA test requires sophisticated manipulation in a central laboratory, and the FOBT has limited diagnostic accuracy due to interference from hemorrhoids. [5, 6]. An ideal cancer screening test should involve easy and non-invasive sampling, show a high correlation with the biology of cancer progression, and have a high detection rate with few false negatives.

MicroRNAs (miRNAs) are a group of short non-coding RNA molecules that are encoded in genomes. Each miRNA negatively regulates hundreds to thousands of protein-coding genes to govern various biological processes, including development, differentiation, and proliferation [7]. Aberrant miRNA expression has been detected in many cancer types [8, 9]. In particular, circulating miRNAs, which are remarkably stable in the bloodstream, and serum miRNA profiles were found to be disease-specific in lung cancer, CRC, and diabetes patients [10]. These natural properties make circulating miRNAs promising biomarkers for disease diagnosis.

Since 2001, more than 500 studies have examined CRC-related miRNAs. Beginning in 2008, some studies began to explore the potential of using miRNAs in blood and stool for the early detection of CRC and the prediction of its prognosis and therapeutic responsiveness (Supplementary references). Most such studies focused on discovering new markers in small sample sets through the use of different methodologies (e.g., microarray or quantitative PCR; qPCR) on individual sample types (e.g., serum, plasma or feces). No previous investigation has used a stepwise approach of gathering the findings from existing studies, assessing each candidate miRNA in various sample types from the same individual, and then evaluating the possible application of certain miRNAs for CRC diagnosis.

In the present study, we comprehensively and critically surveyed the published literature related to CRC-associated miRNAs, selected 46 potential miRNA candidates, and assembled a panel for CRC detection. We established a multiplex RT-qPCR platform and used it to quantify the 46 miRNAs in paired CRC and adjacent normal tissues. We linked the expression level of each miRNA in plasma and stool samples with that in the primary tumor, and selected the most cancer-relevant miRNAs. We then evaluated the performances of the selected miRNAs in retrospective case-control cohort samples. To minimize cost and maximize the clinical applicability, we excluded miRNAs that failed to show any mutual complementary effect in enhancing the rate of cancer detection. Finally, we used a logistic regression algorithm to integrate the expression levels of the selected miRNAs in plasma and stool samples, to aid in sensitively identifying patients at high risk for having CRC.

## RESULTS

### Candidate selection of miRNAs and establishment of a TaqMan RT-qPCR for CRC detection

**Figure 1:**
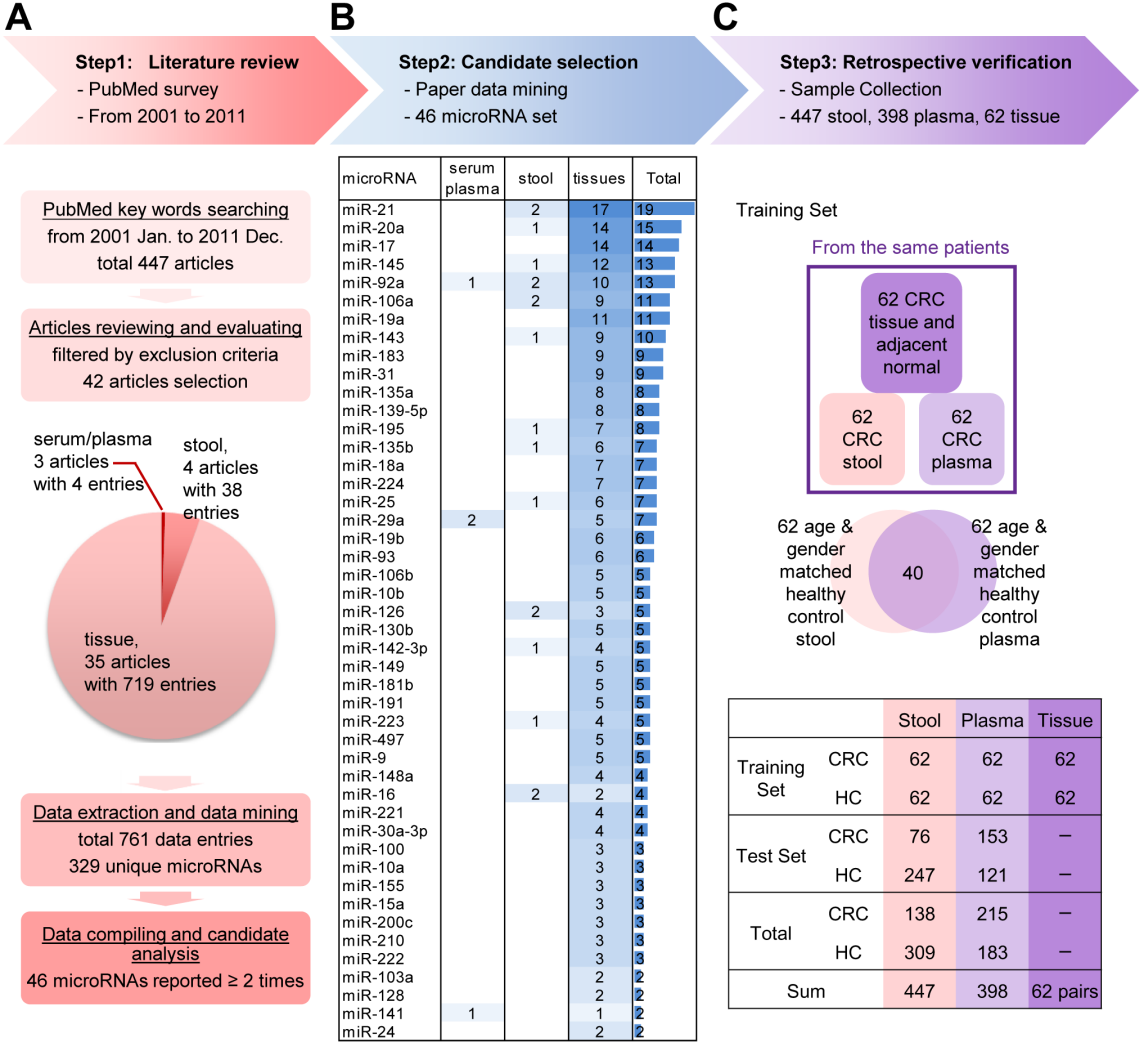
Step-wise strategy for identifying and verifying target miRNAs relevant to colorectal cancer (CRC) detection **A.** A literature review was performed to evaluate reported CRC-related miRNAs from public databases. Articles were filtered using specific criteria, and 42 articles containing 329 unique miRNAs (resolved from 761 data entries) were chosen by detailed filtering criteria (shown in Supplementary Figure 1). Forty-six miRNAs that were reported two or more times in the selected articles were extracted for further study. **B.** The 46 selected miRNAs, ranked by descending number of references (listed in Supplementary Table 1). For example, the most highly cited miRNA, miR-21, was reported by 19 references; 17 demonstrated its presence in tissue samples, and two reported its presence in stool samples. **C.** Verification was performed in retrospectively collected clinical samples with definitive colonoscopy results. Sixty-two paired tumor and normal tissues, 447 stool, and 398 plasma samples were investigated. The samples were divided into two sets: a training set and a test set. The training set included 62 paired tissue, stool and plasma samples obtained from 62 CRC patients, plus 62 age- and gender-matched stool and plasma samples obtained from 84 healthy controls (HCs; including 40 overlapping paired samples). The remaining stool and plasma samples were used as the test set.

To select CRC-related miRNA candidates from the literature, we conducted an extensive review, selected the most frequently reported miRNAs, and verified them with clinical specimens from CRC and control groups, using training and test steps (see Figure 1 for our analytic strategy). First, we systematically evaluated 447 studies through a PubMed search of articles published from January 2001 to December 2011, using keywords such as “CRC miRNA,” “colon cancer miRNA,” and “rectal cancer miRNA.” After excluding papers only investigating the methodologies of miRNAs detection or not focusing on biomarker exploration in human samples, forty-two articles were further examined (the detail exclusion criteria were shown in Supplementary Figure 1). From them, a total of 329 unique miRNAs were obtained and subjected to further data analysis (Figure 1A and Supplementary Figure 1). Most of the data were obtained from tissue samples (35 articles), along with four studies employing stool samples and three involving serum or plasma samples. Real-time PCR (24 articles) and microarray analysis (16 articles) were the techniques most commonly used for miRNA quantitation. From the compiled data, we selected an miRNA candidate set consisting of the 46 most highly reported miRNAs (mentioned from 2 to 19 times) reported to show significant differential expression in serum, stool, or tissue samples (Figure 1B and Supplementary Table 1).

In an effort to develop an miRNA detection method that is easy to implement in the clinical laboratory, we established a quantitation assay that uses multiplex TaqMan stem-loop reverse-transcription (RT)-PCR, with externally spiked-in cel-miR-238 applied as an internal control for between-sample normalization of expression levels. The linearity of the quantitative curves for the 46 miRNAs were evaluated from 5 to 5×10^6^ copies of synthetic templates, and the detection limit was set at a Ct value of 40 (Supplementary Figure 2A). No cross reaction was observed among the PCR primers and TaqMan probes for the 44 miRNAs PCR reactions, with the exception of interference between miR-17 and miR-106a (Supplementary Figure 2B). The efficiency obtained in our one-tube multiplex RT reaction was comparable to those obtained from the 46 individual RT reactions (Supplementary Figure 2C). The duplication precisions obtained using two independent RT reactions or RNA extractions from a single sample were excellent (Supplementary Figure 2D), demonstrating that our miRNA assay is reliable and feasible, and thus may be appropriate for clinical use.

To examine the expression signatures of the 46 selected CRC-relevant miRNAs and their correlations in different sample types, paired tumor/adjacent normal tissue, stool and plasma samples were collected from 62 CRC patients. As controls, 62 stool and plasma samples were collected from 84 age- and gender-matched healthy individuals with negative colonoscopy results. In the training set, the distributions of age and gender were similar between the cancer and control groups (Table 1). Another 323 stool (76 CRC and 247 healthy controls) and 274 plasma (153 CRC and 121 healthy controls) samples were collected to enable us to further verify the discrimination power of the 46 miRNAs in the test set (Figure 1C). In the latter (test) set, the average age in the CRC group was younger than that in control group (Table 1).

**Table 1:** Demographics and sample distribution of colorectal cancer patients and healthy controls in training set and test set

		Stool			Plasma		
		Healthy control	CRC	*p*-value	Healthy control	CRC	*p*-value
Training Set	Sample no	62	62		62	62	
	Age (Mean ± SD)	61.1 ± 3.8	62.3 ± 11.8	0.418	61.0 ± 3.8	62.3 ± 11.8	0.340
	Gender (F/M)	26/36	29/33	0.718	26/36	29/33	0.718
Test Set	Sample no	247	76		121	153	
	Age (Mean ± SD)	47.1 ± 9.4	61.8 ± 10.6	0.000	54.8 ± 4.6	63.9 ± 12.0	0.000
	Gender (F/M)	84/163	31/45	0.338	42/79	70/83	0.083

### Expression profile of 46 miRNAs in paired CRC/normal tissues, stool, and plasma samples of CRC patients

**Figure 2:**
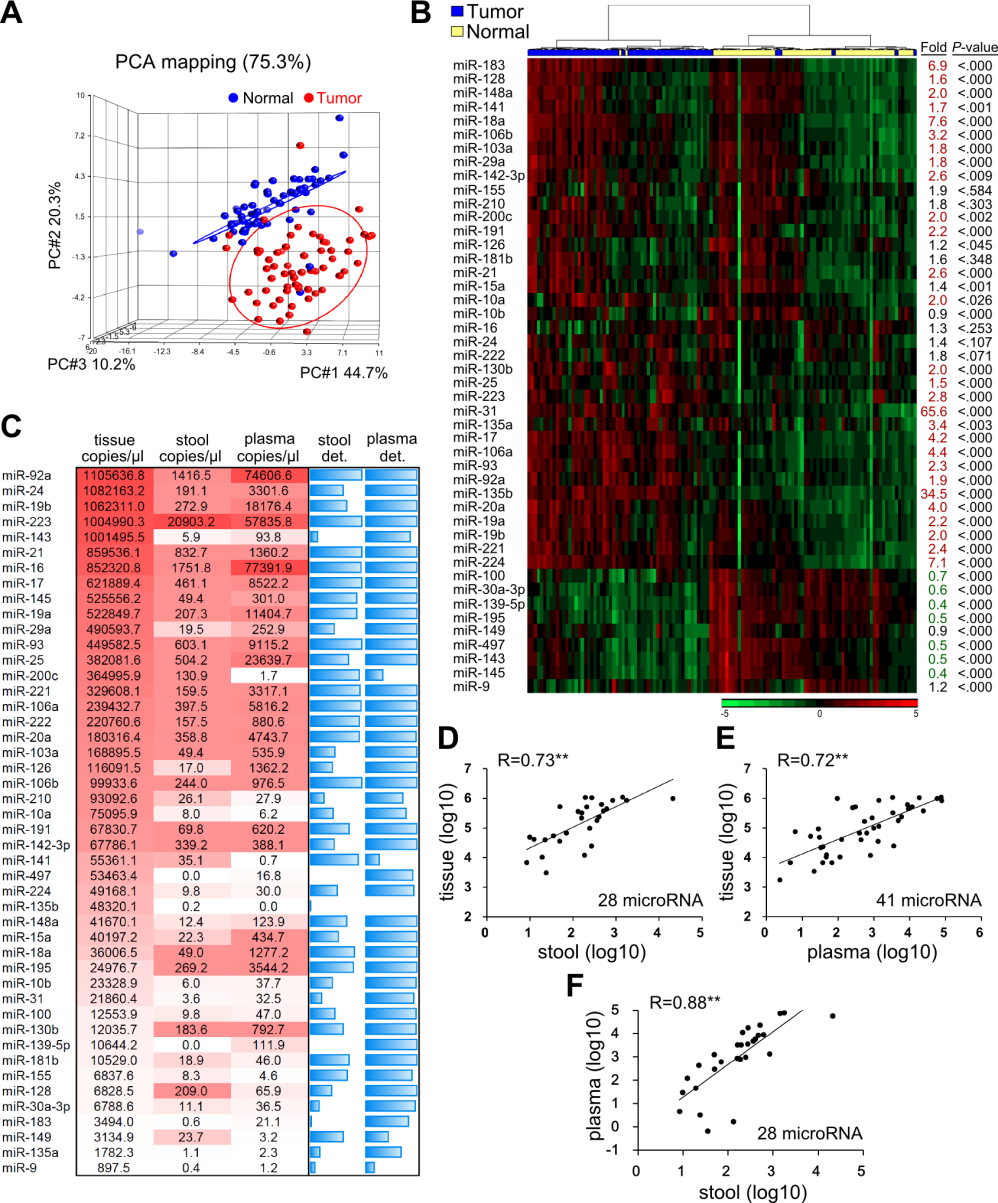
Expression levels of the 46 candidate miRNAs in 62 paired CRC samples **A.** Principle component analysis (PCA) illustrates the correlation between 62 tumors (shown as red dots) and their adjacent normal tissues (blue dots), as defined by the sum effect of the expression levels for the 46 selected miRNAs. **B.** Heatmap representing unsupervised clustering of 62 CRC tumors (blue) and their adjacent normal tissues (yellow) according to the expression levels of the 46 miRNAs. Fold was defined as the ratio of the average expression level of a specific miRNA in the 62 tumors versus those in the corresponding normal tissues. P was calculated by paired *t*-test. **C.** MiRNA expression levels in paired tumor tissues, stool, and plasma samples from 62 CRC patients, shown as the average copy number of each miRNA per microliter of the three sample types. MiRNA quantities are presented as a color gradient, and ranked according to their concentrations in tumor tissues. The two blue columns depict the detection rate (det.) of specific miRNAs in 62 stool and plasma samples. Detection rate was defined as the percentage of samples with Ct < 40 after normalization with respect to spiked cel-miR-238 and full blue bar stands for 100% detection rate. **D.**-**F.** Correlation of “detectable” miRNAs among the three sample types. In this case, “detectable” was defined as the detection rate of a given miRNA > 50% among 62 CRC patients. Twenty-eight miRNAs in stool and 41 miRNAs in plasma were classified as “detectable” using this criterion. Each dot indicates the average concentration of an individual miRNA in the 62 samples, given on log scale. Correlation coefficients (R) are shown (**, *P <* 0.001).

The levels of the 46 candidate miRNAs were measured in 62 paired tumor and adjacent normal tissues. Principle of component analysis (PCA) revealed that the expression patterns of the miRNAs in tumor tissues could be easily distinguished from those in their normal counterparts (Figure 2A). Compared to normal tissues, tumor tissues exhibited 37 remarkably up-regulated miRNAs (increased from 1.2- to 65.6-fold) and nine significantly down-regulated miRNAs (decreased from 0.9- to 0.3-fold) (Figure 2B). These findings confirmed that our 46 selected miRNA could be considered candidate CRC biomarkers.

We also compared the expression levels of the 46 miRNAs in stool and plasma samples obtained from the same patients. Interestingly, when we ranked the expressions of these miRNAs based on the average copy number across the 62 samples, the rankings were similar across the three specimen types (Figure 2C). MiRNA-92a, miR-223, and miR-16 were the most relatively abundant miRNAs in all three samples types. However, some miRNAs revealed large between-sample-type fluctuations in concentration. In general, miRNA levels in stool specimens were at least 2-log lower than those in tumor tissues. This issue might hamper their clinical utility due to limited detection accuracy. After we discarded any miRNA that had a low expression level and was undetectable in over 50% of measured samples, 28 miRNAs in stool and 41 miRNAs in plasma were retained as “detectable” miRNAs for further investigation. As shown in Figure 2D-2F, these detectable miRNAs showed high expressional correlations between tissue and stool (*R* = 0.73), tissue and plasma (*R* = 0.72), and plasma and stool (*R* = 0.88). This rationalizes that such circulating miRNAs might be clinically applicable for CRC detection.

### Two common miRNAs with the best performance in stool and plasma were selected after validation in the total dataset

**Figure 3:**
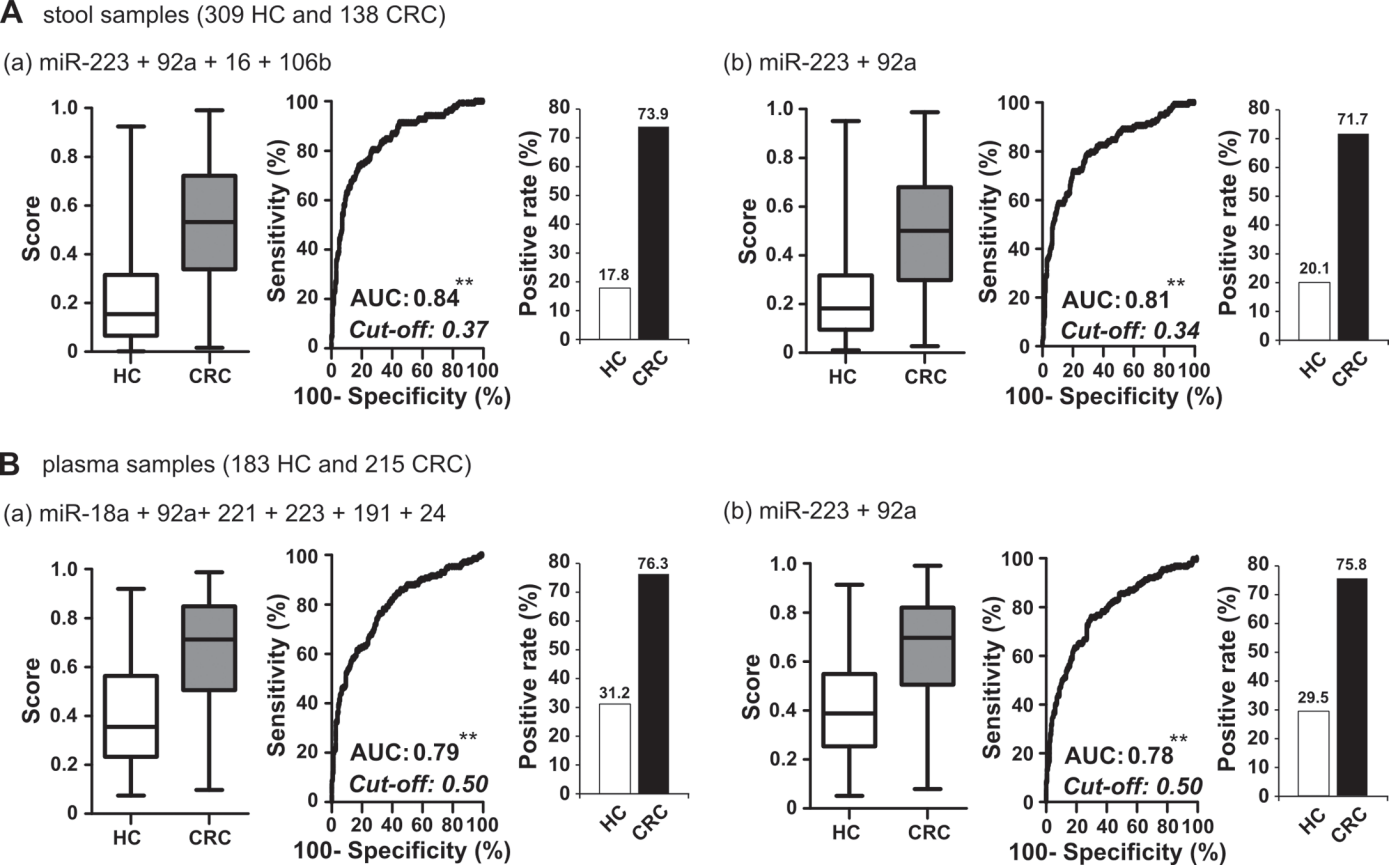
Comparison of clinical performance between stool-related miRNAs and plasma-related miRNAs selected from test set and two common miRNAs **A.** In total stool samples, logistic regression was used to integrate the summed effects of (a) four miRNAs or (b) the two common miRNAs in discriminating between healthy control and cancer samples. Scores ranging from 0 to 1 were generated for each sample; the whisker box plot denotes the distribution of scores in the healthy control and CRC groups. The positive rate in each group was calculated according to the cut-off value obtained using Youden's index on the ROC curve. **B.** In total plasma samples, the sum effect of (a) six miRNAs or (b) the two common miRNAs in discriminating between healthy control and cancer samples (**, *P* < 0.001).

The mean level, fold-change and area under the curve (AUC) for the receiver-operating characteristic (ROC) curve of 28 detectable miRNAs in stool and 41 detectable miRNAs in plasma for discriminating CRC patients from healthy controls in training set were calculated. Table 2A lists the five and eleven miRNAs that showed significantly altered expression levels with fold-changes larger than 2 or less than 0.5 and with discriminating power of AUC values larger than 0.7 between CRC and healthy control groups in stool and plasma samples, respectively. Among them, miRNA-223 had a 16.55-fold higher expression level in CRC stool samples than that in healthy controls, and miR-18a and miR-92a had AUC values > 0.8 for the ability of plasma samples to distinguish cancer samples from healthy control samples. Then, the five and eleven miRNAs selected from the training set were subsequently quantified and validated in a larger independent test set. Among them, four and six miRNAs maintained not only their up-regulation trends but also discriminating powers (AUC > 0.7) in the cancer cases, as assessed in 323 stool and 274 plasma samples (Table 2B). In addition, miR-223 and miR-92a are two common miRNAs that exhibited fair performance in both stool and plasma samples. To elucidate whether the four validated miRNAs in stool samples and six validated miRNAs in plasma samples could have additive effects for identifying CRC patients, we conducted logistic regression in the total sample sets of stool and plasma. Figure 3 demonstrates the clinical performances of the combination of four stool-related miRNAs or six plasma-related miRNAs or just the two common miRNAs, miR-223 and miR-92a, for detecting CRC in total 447 stool and 398 plasma samples, respectively. Logistic regression analysis gave different weighted constants for each recruited miRNA and generated a sum score for each sample. The detection rate of CRC and the false positive rate in the healthy control group were calculated by using the ROC analysis to set an optimal cutoff with maximum sensitivity and specificity. Notably, we did not observe any significant increase of sensitivity or specificity for CRC detection when we employed the multiple miRNA combination strategy, compared to the result obtained using the two common miRNAs (sensitivity and specificity: 73.9% and 82.2% vs. 71.7% and 79.9%, respectively, in stool; and 76.3% and 68.8% vs. 75.8% and 70.5%, respectively, in plasma). Therefore, we decided to use the common miRNAs, miR-223 and miR-92a, as our stool and plasma panel for further analysis.

**Table 2:** Selected miRNAs with significantly differential expression between CRC and healthy control groups in training set A) and test set B). Gray block in test set highlights the same performance trend of miRNAs with >2 fold-change and >0.7 AUC represented in training set

A. Training set	miRNA	Healthy control (mean±SD)	CRC (mean±SD)	Fold-change	*p*-value (*t*-test)	AUC	95% CI	*p*-value
stool (H/C=62/62)	miR-223	6.53 ± 3.86	10.58 ± 3.56	16.55	0.000	0.787	0.705-0.869	0.000
	miR-92a	7.27 ± 1.97	9.07 ± 2.17	3.48	0.000	0.739	0.651-0.828	0.000
	miR-16	5.79 ± 2.58	7.93 ± 2.89	4.40	0.000	0.726	0.634-0.818	0.000
	miR-20a	3.82 ± 2.77	5.89 ± 2.77	4.20	0.000	0.717	0.626-0.807	0.000
	miR-106b	5.02 ± 2.14	6.79 ± 2.19	3.41	0.000	0.713	0.623-0.804	0.000
plasma (H/C=62/62)	miR-18a	8.00 ± 1.45	9.97 ± 1.23	3.92	0.000	0.855	0.789-0.920	0.000
	miR-92a	14.08 ± 0.98	15.60 ± 1.32	2.85	0.000	0.833	0.763-0.904	0.000
	miR-221	10.37 ± 1.12	11.40 ± 1.14	2.04	0.000	0.750	0.663-0.836	0.000
	miR-497	4.75 ± 1.50	3.16 ± 1.85	0.33	0.000	0.743	0.657-0.830	0.000
	miR-141	1.71 ± 2.24	0.32 ± 0.67	0.38	0.000	0.735	0.646-0.823	0.000
	miR-223	13.91 ± 1.49	15.24 ± 1.45	2.51	0.000	0.734	0.646-0.823	0.000
	miR-191	6.95 ± 1.22	8.24 ± 1.57	2.45	0.000	0.734	0.646-0.822	0.000
	miR-24	10.22 ± 1.11	11.25 ± 1.35	2.05	0.000	0.734	0.645-0.823	0.000
	miR-10b	5.86 ± 1.14	4.86 ± 1.31	0.50	0.000	0.726	0.636-0.815	0.000
	miR-25	12.80 ± 1.15	13.83 ± 1.52	2.04	0.000	0.712	0.620-0.803	0.000
	miR-31	5.31 ± 1.35	3.92 ± 2.02	0.38	0.000	0.710	0.617-0.802	0.000

B. Test set	miRNA	Healthy control (mean±SD)	CRC (mean ± SD)	Fold-chande	*p*-value (*t*-test)	AUC	95% CI	*p*-value
stool (H/C=247/76)	miR-223	5.98 ± 2.86	9.43 ± 3.39	10.91	0.000	**0.796**	0.734-0.858	0.000
	miR-92a	7.07 ± 2.11	9.10 ± 2.11	4.10	0.000	**0.748**	0.683-0.814	0.000
	miR-16	5.7 ± 1.72	7.46 ± 2.82	3.38	0.000	**0.703**	0.630-0.777	0.000
	miR-20a	3.96 ± 2.1	5.16 ± 2.80	2.30	0.001	0.639	0.561-0.716	0.000
	miR-106b	5.48 ± 1.97	7.06 ± 2.25	3.00	0.000	**0.705**	0.637-0.772	0.000
plasma (H/C=121/153)	miR-18a	7.86 ± 1.23	9.05 ± 1.65	2.28	0.000	**0.715**	0.656-0.775	0.000
	miR-92a	13.84 ± 0.91	14.88 ± 1.25	2.06	0.000	**0.751**	0.693-0.808	0.000
	miR-221	10.41 ± 0.98	11.50 ± 1.42	2.13	0.000	**0.743**	0.684-0.801	0.000
	miR-497	4.75 ± 1.34	4.37 ± 1.63	0.77	0.040	0.576	0.508-0.643	0.031
	miR-141	1.71 ± 2.11	1.13 ± 1.44	0.67	0.011	0.581	0.513-0.650	0.021
	miR-223	13.9 ± 1.12	14.96 ± 1.65	2.09	0.000	**0.707**	0.646-0.768	0.000
	miR-191	6.84 ± 1.08	7.88 ± 1.47	2.05	0.000	**0.714**	0.654-0.775	0.000
	miR-24	9.99 ± 0.9	11.10 ± 1.45	2.16	0.000	**0.753**	0.696-0.811	0.000
	miR-10b	6.17 ± 1.17	5.34 ± 1.80	0.56	0.000	0.645	0.581-0.710	0.000
	miR-25	13.1 ± 0.87	13.65 ± 1.29	1.46	0.000	0.642	0.577-0.708	0.000
	miR-31	5.41 ± 1.25	5.04 ± 1.61	0.77	0.040	0.559	0.491-0.627	0.092

### Complementary effect of miR-223 and miR-92a in stool and plasma enhances the CRC detection rate

**Figure 4:**
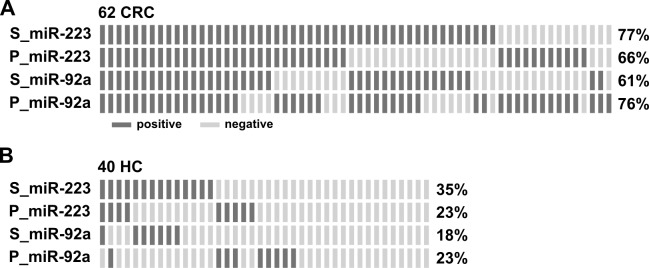
Complementary effect of measuring miR-223 and miR-92a in individual stool and plasma samples for detection of CRC **A.** and **B.** Distribution of positive cases (dark bar) identified by the expression levels of miR-223 and miR-92a in stool (S_) and plasma (P_) in 62 CRC patients (A) and 40 healthy controls who had paired stool and plasma samples available (B). The positive rate for each miRNA was defined using a cut-off calculated from Youden's index of the ROC.

**Figure 5:**
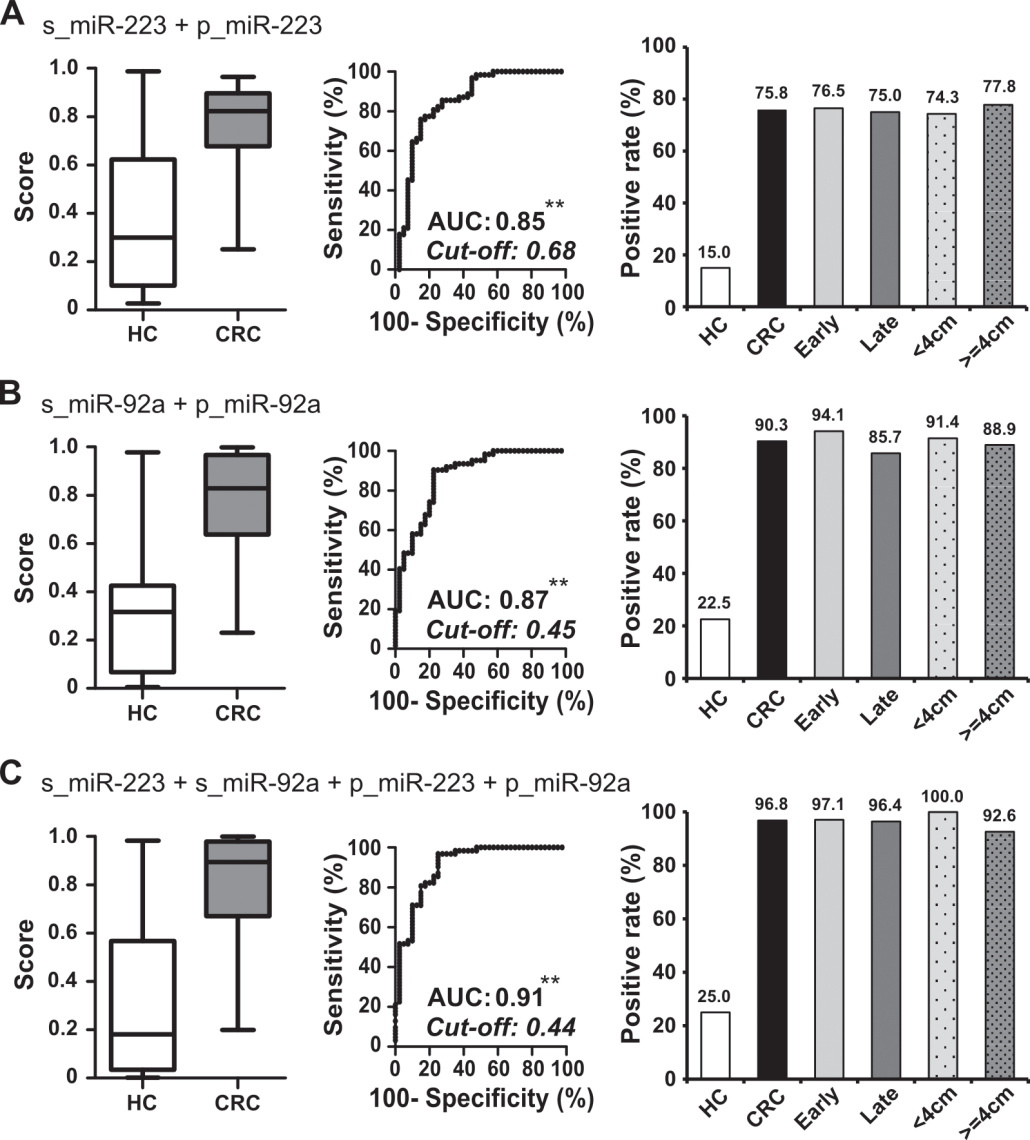
Additive detection of cancer is achieved by integrating measurements of miR-223 and miR-92a in paired stool and plasma samples **A.**-**C.** To evaluate the performance of different combinations of miRNAs in paired samples, we used logistic regression to integrate the sum effect of examining miR-223 (A), miR-92a (B), and miR-223 plus miR-92a (C) in both stool and plasma samples. The whisker box plot denotes the distribution of scores in 40 healthy controls and 62 CRC patients; the positive rates were calculated according to the cut-off value determined by Youden's index for the ROC curve (**, *P* < 0.001). The CRC group was further divided into subgroups based on the tumor stage and tumor size, and positive rates were compared between subgroups. Tumors of AJCC stages I and II were defined as early stage (*n* = 34, light gray bar), while all others were considered late stage (*n* = 28, heavy gray bar). Tumor size was taken as the largest measurement among the length, depth, and height of the tumor. When we used 4 cm as the cutoff, there were 35 tumors < 4 cm (spotted light gray bar) and 27 tumors >= 4 cm (spotted heavy gray bar).

To further clarify if there could be a complementary effect of detecting miR-223 and miR-92a in the stool and plasma samples of a single patient, we investigated their expressional distribution in 62 CRC patients and 40 healthy controls for whom paired stool and plasma samples were available. Figure 4 illustrates the positive rates of the two markers in the two sample types, based on their individual ROC-derived cutoffs, and their repulsive distributions in the cancer and healthy control groups. The use of miR-223 in stool yielded a 77% detection rate for CRC. The additional analysis of miR-223 in plasma and miR-92a in stool and plasma samples improved detection to 100%; however, a 60% of high false-positive rate was observed in healthy controls. To further investigate the best clinical performance by possible combination of miRNAs in two types of specimen by taking advantage of complementary roles of two common miRNAs, we applied a single marker (miR-223 or miR-92a) or our combined marker analysis (miR-223 + miR-92a) in stool and plasma samples to examine the clinical effectiveness of CRC detection, as assessed by logistic regression. Figure 5 showed the combined performance of miR-223 and miR-92a in the two specimen types yielded a sensitivity of 96.8% without much loss of specificity, which remained relatively high at 75%. Furthermore, if we divided the 62 CRC patients into two subgroups based on their tumor stage and tumor size and compared the positive rates of miR-223 in stool and plasma, miR-92a in stool and plasma, and miR-223+miR-92a in stool and plasma between the groups, we found that CRC patients with early stage disease and/or smaller tumor size yielded results comparable to those of patients with late stage disease and/or larger tumor size. This demonstrates that our miRNA panel could feasibly be used for the early detection of CRC.

## DISCUSSION

In the present study, we collected a myriad of CRC-relevant miRNA biomarkers published in the literature and explored their clinical applicability for CRC detection. Forty-six miRNAs were carefully selected, and a multiplex RT-qPCR test was successfully developed to quantify the expression levels of these miRNAs in non-invasively obtained specimens. Using paired-tissue, stool and plasma samples from 62 CRC patients, we show for the first time that there is a strong correlation of the relative concentrations of these 46 miRNAs in the three specimen types. Using case-control cohort samples, we found that two common miRNAs, miR-223 and miR-92a, performed best as biomarkers for detecting CRC. This suggests the novel concept that the detectable miRNAs in stool and plasma samples may be complementary in their relevance as clinical biomarkers.

Numerous markers have been reported for CRC detection, but few have moved into clinical application. One of the major concerns is whether the biomarkers are derived from the CRC tumors. Our present study solves this by: (1) establishing the expression profiles of the 46 candidate miRNAs and showing that they can be used to discriminate CRC tumors versus adjacent normal tissues, which suggests that these miRNAs may be strongly associated with CRC carcinogenesis; (2) showing that the expression levels of these miRNAs are correlated in paired-tissue, stool, and plasma samples from the same patients, which supports the feasibility of detecting CRC by assessing such specimens for the selected miRNAs; and (3) validating our method in clinical samples that are convenient to access in the current clinical setting, which provides evidence that our technique could be moved into clinical application. MiR-223 and miR-92a were initially selected as candidates because they were previously demonstrated to participate in CRC carcinogenesis, and have been reported as diagnostic biomarkers in either stool or plasma. MiRNA-223 is an onco-miRNA; it has been shown to target survival- and death-related genes, and its knockdown was shown to decrease cell proliferation, migration and invasion in CRC cells [11, 12]. MiRNA-92a also functions as an oncogene *via* an interaction with its target, PTEN [13]. Several recent studies determined the levels of miR-223 and miR-92a in blood [14, 15] or stool [16-18] in CRC patients, and obtained results consistent with our present findings. Moving beyond these studies, we integrated the detection ability of these two miRNAs when applied in both stool and plasma samples, and showed that they offer an additive power that increases the rate of CRC diagnosis. This complementary effect may reflect the heterogeneity of individual tumors and the biological roles of these two miRNAs. Further studies are warranted.

As miRNAs may be secreted from the tumor site into the bloodstream *via* exosomes or apoptotic processes [19], high background noise (in terms of miRNAs) may arise from blood cells themselves [20, 21] or from other organs. A previous study found that miR-223 is the major miRNA in neutrophils [22], which could challenge our ability to state that this miRNA level in blood is correlated with cancer. However, we investigated this possible interference and found that there was no evidence of correlation between the expression level of miR-223 in plasma and the white blood cell or neutrophil count in our study population (Supplementary Figure 3). On the other hand, the miRNAs in stool are likely to arise from exfoliated colonocytes and thus will more directly reflect the biological changes in lesions of the intestinal lumen [23]. This could explain the larger fold-changes seen for miR-223 and miR-92a in stool samples (16.55- and 3.48-fold) compared to those in plasma (2.51- and 2.85-fold) when we compared the CRC patients and control group in our study.

The amount and stability of miRNAs are two major factors that contribute to the success of miRNA measurements, especially in stool samples. We found that the concentrations of detectable miRNAs were 10- to 100-fold lower in plasma and stool compared to the sampled tissues. Thus, it is important to select more abundant (not necessarily the most differentially expressed) miRNAs when seeking to identify biomarkers. Wu's group recently reported that miR-31 and miR-135b were the most highly upregulated miRNAs in CRC tissues, and that miR-135b in stool yielded a CRC detection rate of 76% [24]. These two miRNAs were also strongly enhanced in the tumor tissues of the present study (65.6-fold and 34.5-fold, respectively, compared to paired normal tissues; Figure 2B). However, they were not the most abundant miRNAs in the tumor tissues, and they were almost undetectable in our stool samples (Figure 2C). This apparent discrepancy might reflect the amount of stool processed for the measurement of miRNAs: 200∼300 mg of fresh stool was used in Wu's study, whereas we used 300 μL of residual stool from FOBT tubes. For clinical practice, it is more convenient to use fixed volumes of buffered stool than to manipulate scaled quantities of fresh stool. Previous studies also suggest that miRNAs are stable in FOBT buffer for up to 5 days when stored at 4°C [25]. Using FOBT tube-collected stool samples, our present cohort study demonstrates that the abundant miRNAs, miR-223 and miR-92a, are good targets for miRNA quantitation in stool.

Under most circumstances, age is a major determinant for cancer screening; the elderly usually have a higher positive rate, and should be given priority for screening [26]. In our study, the age distribution of the healthy control group in the test set was significantly lower than that in CRC group. To elucidate the effect of age on the expression of cancer-related miRNAs, we used Spearman's correlation on our total datasets for stool and plasma. This analysis demonstrated that there was no significant correlation between age and miRNA levels in stool or plasma samples (Supplementary Table 2) and further strengthened the appropriateness for the selection of miRNAs under the age mismatch condition.

Compared to other available non-invasive CRC screening tests, the 96.8% sensitivity obtained by examining the combination of miR-223 and miR-92a in stool and plasma is superior to those obtained using the FOBT (around 70∼75% [4]) or the multi-target stool DNA test (92.3% [27]). However, the lower specificity (75%) of our miRNA panel could result in more individuals undergoing unnecessary colonoscopies to rule out CRC. To address this, the incorporation of a high-specificity test, such as FOBT (95% specificity), could help exclude low-risk individuals. A similar strategy was incorporated into the multi-target stool DNA test developed by Exact Science, and successfully elevated the specificity to 86.6% [27]. An ideal cost-effective cancer screening test would involve a limited number of biomarkers and yield a high detection rate at an early stage of cancer. Thus, we validated the efficacy of a two-miRNA panel for screening both stool and plasma instead of using the top four miRNAs in stool and the top six miRNAs in plasma. This makes the panel simpler and more affordable for clinical application. The two-miRNA panel yields a high degree of sensitivity at an early stage of CRC and tumors with small size (Figure 6), which is critical for early detection of this cancer in the general population.

In summary, we herein propose and support the use of a two-miRNA panel consisting of miR-223 and miR-92a. Quantitation of these two miRNA levels in both stool and plasma can cost effectively yield a high detection rate for CRC. To further validate the effectiveness of this miRNA panel in the future, an independent prospective cohort of patients with advanced polyps should be recruited.

## MATERIALS AND METHODS

### Study groups

This study included 291 patients with CRC diagnosed between February 2012 and October 2013 at Chang Gung Memorial Hospital in Taiwan. Patients with family history and concomitant tumors were excluded. Tumors were staged according to the 2009 American Joint Committee on Cancer (AJCC) staging criteria (7th edition) [28]. Pathological parameters were also recorded, including the tumor location and size. Patients were divided into the training and test subgroups. Sixty-two CRC patients with available paired tumor and adjacent normal tissue samples (resected during operation) and their corresponding stool and plasma samples (collected prior to surgery) were designated as the training group. The four specimens from each CRC patient in the training group were used to investigate the individual-level correlations of expression levels among candidate miRNAs and to examine the clinical performance of one miRNA in various sample types. The remaining 229 patients were used as the test group. Of them, 76 patients were asked to provide stool samples, and 153 separately provided plasma samples during routine blood testing upon admission. There was no overlap of sample types in the test group.

For the control group, we recruited 452 volunteers from the Health-Check Center at Chang Gung Memorial Hospital. All participants were self-reported as negative for any colorectal cancer history or inflammatory bowel disease, and reported having negative colonoscopy results (defined by an absence of neoplasia or advanced polyps or only the presence of polyps with pathologically confirmed hyperplasia and < 10 mm tubular adenoma). Due to the younger age of the control group, we tentatively selected 84 elder healthy individuals to match the age and gender distribution in the CRC training group. Among them, 40 participants provided both stool and plasma specimens; these paired normal samples were used to evaluate the specificity of the combined use of miRNAs in stool and plasma. The remaining participants were placed in the test group; 247 provided stool samples and 121 provided plasma samples, with no overlap. All patients and healthy individuals provided written informed consent. The study was approved by the institutional review board of Chang Gung Memorial Hospital (IRB approval no: 100-4602B).

### Sample collection

EDTA blood or fresh stool samples were collected from CRC patients prior to bowel preparation and surgery. Blood samples were centrifuged at 2000*g* for 10 min, and the obtained plasma was aliquoted into several tubes for further use. Stool samples were dipped with a designated FOBT swab and inserted into preservation buffer in FOBT tube (Eiken Chemical, Tokyo, Japan). After being vortexed thoroughly, buffered stool samples were squeezed out of the device and collected for storage. Fresh tumor and adjacent normal (at least 5 cm away from the tumor) tissue samples were taken from CRC patients during surgery. In the control groups, EDTA-plasma samples left over from the routine complete blood count test and residual samples from the FOBT were collected within 4 h after routine laboratory examination. All samples were stored at −80°C until use.

### RNA extraction

Tissue samples (2 mm^3^) were placed in tubes containing QIAzol Lysis Reagent and 7-mm stainless steel beads (Qiagen, CA, USA) and homogenized using a TissueLyser II (Qiagen) at 30 Hz until the tissue debris became invisible. Plasma samples with a hemolysis titer higher than “trace,” as inspected by the color chart, were discarded. Plasma and stool samples were subjected to high-speed centrifugation at 12000*g* for 5 min to remove debris. 300 μL supernatants of centrifuged stool and plasma samples were subsequently added with 10^7^ copies of synthetic RNA corresponding to *Caenorhabditis elegans* miR-238 (5′-UUU GUA CUC CGA UGC CAU UCA GA-3′) (IDT, Coralville, IA) as the spike-in control and 700 μL QIAzol Lysis Reagent. Total RNA was extracted from above pretreated stool, plasma, or homogenized tissue samples by miRNeasy Mini Kit according to the manufacturer's protocol (Qiagen). The obtained total RNA was dissolved in 30°C μL RNase-free water and stored at −80°C.

### Reverse transcription

To quantify miRNA, we used the stem-loop reverse transcription-polymerase chain reaction (RT-PCR) principle [29] and miRNA sequences obtained from miRBase (http://www.mirbase.org). Tissue RNA (500 μg), stool RNA or plasma RNA (5.4 μL each) were subjected to RT using a TaqMan miRNA Reverse Transcription Kit and TaqMan^®^ MicroRNA Assays (Applied Biosystems, Foster City, CA) according to a modified version of the manufacturer's protocol. Briefly, RT primers corresponding to the 47 miRNAs from TaqMan^®^ MicroRNA Assays (Applied Biosystems, Foster City, CA) were mixed together to convert the miRNAs into their corresponding cDNAs in a single reaction, and individual PCR was performed using the following cycling conditions: 16°C for 30 min, followed by 50 cycles at 20°C for 30 s, 42°C for 30 s, and 50°C for 1 s, and a final step of 70°C for 10 min. The products were diluted 5-fold with 0.1X TE buffer prior to quantitative PCR (qPCR).

### Quantitative polymerase chain reaction

Quantification of individual miRNAs was performed with the TaqMan Human MiRNA Assay (Applied Biosystems) according to the manufacturer's instructions, using a QuantStudio^™^ 12K flex real-time PCR system (Applied Biosystems). After qPCR, the Ct value of each miRNA was normalized to that of the spike-in control, cel-miR-238. The copy numbers of individual miRNAs were calculated from a standard curve plotted using 5 to 5 × 10^6^ copies of synthetic cDNA oligonucleotides. The analytical detection limit was defined as a Ct value of 40; miRNAs with Ct > 40 were considered undetectable.

### Data processing and statistical analysis

This was a cross-sectional case-control cohort study. Descriptive statistics are summarized with means, standard deviations and percentages. Intergroup comparisons were conducted by paired *t* tests and Mann-Whitney tests, and correlations between two groups were assessed by Spearman correlation. All statistical analyses, including plotting of whisker boxes, calculation of the area under the curve (AUC) of the receiver operating characteristic (ROC) curve for specific miRNA, and logistic regression, were conducted using PASW Statistics 18.0 (SPSS Inc., Chicago, IL). A *P-*value less than 0.05 (two-tailed) was considered statistically significant. A heatmap of miRNA expression in CRC tumor and adjacent normal tissues was constructed using non-supervised hieratical clustering and principal component analysis (PCA), as applied with Partek Genomics Suite 6.6 (Partek Inc, USA).

## SUPPLEMENTARY MATERIAL FIGURES AND TABLE


