# Supplementary material for: MicroRNA-223 and microRNA-92a in stool and plasma samples act as complementary biomarkers to increase colorectal cancer detection
Source: Oncotarget. 2016 Feb 1;7(9):10663–75. doi: 10.18632/oncotarget.7119 (PMC4891149; doi:10.18632/oncotarget.7119)
Supplement: Supplementary file 1 [file oncotarget-07-10663-s001.pdf]

MicroRNA-223 and microRNA-92a in stool and plasma samples act as complementary biomarkers to increase colorectal cancer detection

Supplementary Material

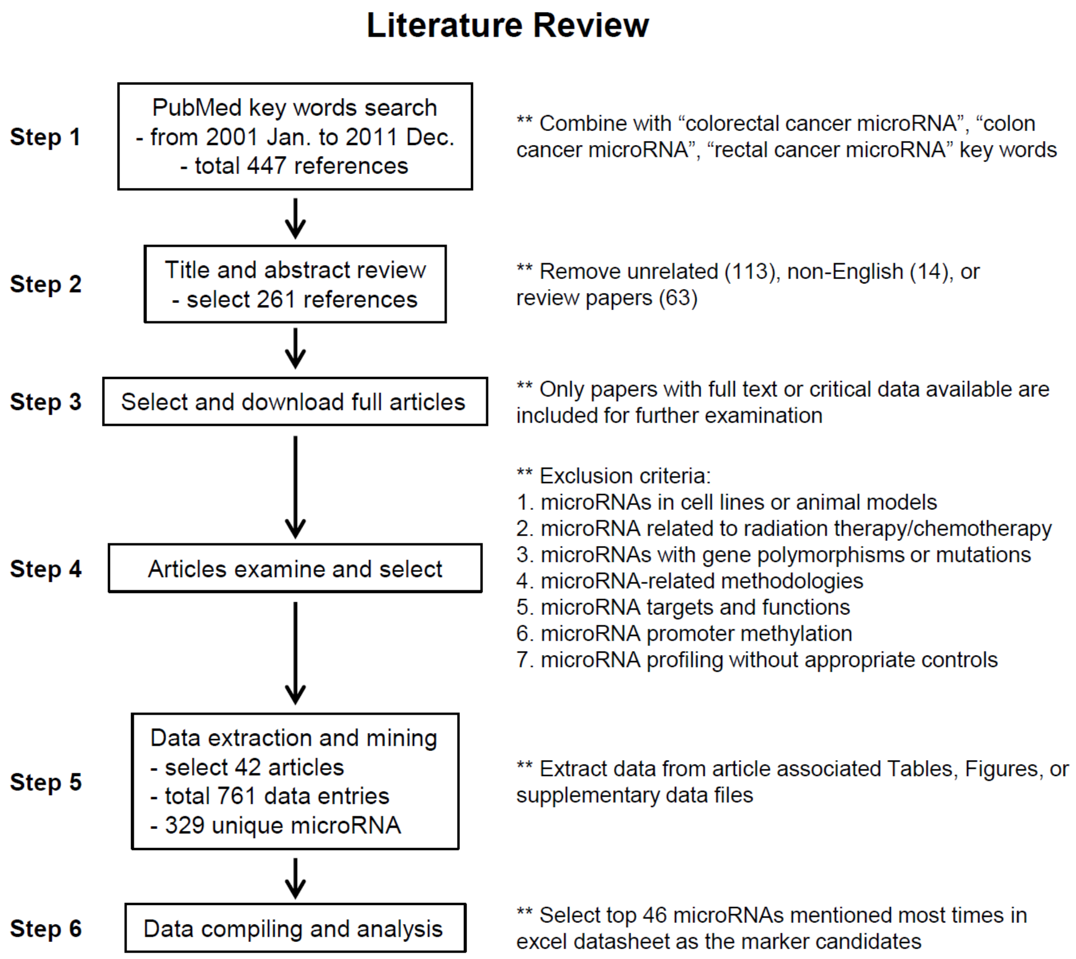

Supplementary Figure 1. The flowchart of literature review listed 6 steps and criteria for selection of 46 CRC-related miRNAs.

Supplementary Table 1. Summary of the references for individual miRNA. Total 42 references are numbered and listing below the table.

microRNA	Total	References
miR-21	19	2,3,5,6,9,10,13,16,19,20,21,26,27,30,32,36,39,40,42
miR-20a	15	2,3,6,8,10,11,13,15,16,20,23,30,32,36,40
miR-17	14	2,6,7,8,10,11,13,15,16,19,23,30,32,36
miR-145	13	1,3,5,9,10,11,11,16,19,20,21,23,32
miR-92a	13	3,6,9,10,11,15,17,19,20,23,30,35,38
miR-106a	11	2,3,6,7,8,10,13,16,20,26,35
miR-19a	11	3,8,10,15,16,19,22,30,36,37,40
miR-143	10	1,5,10,16,19,20,21,23,27,39
miR-183	9	3,11,16,18,19,23,32,32,36
miR-31	9	3,5,11,16,18,23,36,37,39
miR-135a	8	3,6,8,19,23,30,37,42
miR-139-5p	8	3,10,16,18,19,36,37,39
miR-195	8	10,16,19,29,32,32,38,39
miR-135b	7	3,18,19,22,30,36,38
miR-18a	7	10,11,15,16,19,22,30
miR-224	7	3,11,16,18,22,32,40
miR-25	7	8,10,13,16,23,32,38
miR-29a	7	3,8,11,16,17,35,41
miR-19b	6	15,16,19,30,35,36
miR-93	6	6,10,16,23,32,36
miR-106b	5	6,10,13,16,22
miR-10b	5	10,16,18,19,39
miR-126	5	7,19,20,28,38
miR-130b	5	8,10,16,32,36
miR-142-3p	5	8,9,10,36,38
miR-149	5	3,10,12,36,37
miR-181b	5	3,4,6,10,16
miR-191	5	2,4,8,23,35
miR-223	5	2,6,23,32,38
miR-497	5	16,18,32,37,39
miR-9	5	2,3,12,18,37
miR-148a	4	3,8,24,36
miR-16	4	10,20,23,38
miR-221	4	2,8,10,36
miR-30a-3p	4	3,6,16,18
miR-100	3	3,10,19
miR-10a	3	2,8,10
miR-155	3	2,3,10
miR-15a	3	3,8,36
miR-200c	3	3,4,10
miR-210	3	3,6,10
miR-222	3	6,8,10
miR-103a	2	8,10
miR-128	2	8,10
miR-141	2	10,33
miR-24	2	2,40

1. Michael MZ, O' Connor SM, van Holst Pellekaan NG, Young GP, James RJ. Reduced accumulation of specific microRNAs in colorectal neoplasia. Mol Cancer Res. 2003 Oct;1(12):882-91.
2. Volinia S, Calin GA, Liu CG, Ambs S, Cimmino A, Petrocca F, Visone R, Iorio M, Roldo C, Ferracin M, Prueitt RL, Yanaihara N, Lanza G, Scarpa A, Vecchione A, Negrini M, Harris CC, Croce CM. A microRNA expression signature of human solid tumors defines cancer gene targets. Proc Natl Acad Sci U S A. 2006 Feb 14;103(7):2257-61. Epub 2006 Feb 3.
3. BandrÃCs E, Cubedo E, Agirre X, Malumbres R, ZÃ¡rate R, Ramirez N, Abajo A, Navarro A, Moreno I, MonzÃ³ M, GarcÃ-a-Foncillas J. Identification by Real-time PCR of 13 mature microRNAs differentially expressed in colorectal cancer and non-tumoral tissues. Mol Cancer. 2006 Jul 19;5:29.
4. Xi Y, Formentini A, Chien M, Weir DB, Russo JJ, Ju J, Kornmann M, Ju J. Prognostic Values of microRNAs in Colorectal Cancer. Biomark Insights. 2006;2:113-121.
5. Slaby O, Svoboda M, Fabian P, Smerdova T, Knoflickova D, Bednarikova M, Nenutil R, Vyzula R. Altered expression of miR-21, miR-31, miR-143 and miR-145 is related to clinicopathologic features of colorectal cancer. Oncology. 2007;72(5-6):397-402. Epub 2008 Jan 15.
6. Schetter AJ, Leung SY, Sohn JJ, Zanetti KA, Bowman ED, Yanaihara N, Yuen ST, Chan TL, Kwong DL, Au GK, Liu CG, Calin GA, Croce CM, Harris CC. MicroRNA expression profiles associated with prognosis and therapeutic outcome in colon adenocarcinoma. JAMA. 2008 Jan 30;299(4):425-36.
7. DÃ-az R, Silva J, GarcÃ-a JM, Lorenzo Y, GarcÃ-a V, PeÃ±a C, RodrÃ-guez R, MuÃ±oz C, GarcÃ-a F, Bonilla F, DomÃ-nguez G. Deregulated expression of miR-106a predicts survival in human colon cancer patients. Genes Chromosomes Cancer. 2008 Sep;47(9):794-802.
8. Monzo M, Navarro A, Bandres E, Artells R, Moreno I, Gel B, Ibeas R, Moreno J, Martinez F, Diaz T, Martinez A, BalaguÃ© O, Garcia-Foncillas J. Overlapping expression of microRNAs in human embryonic colon and colorectal cancer. Cell Res. 2008 Aug;18(8):823-33.
9. Schepeler T, Reinert JT, Ostenfeld MS, Christensen LL, Silahtaroglu AN, DyrskjÃ¸t L, Wiuf C, SÃ¸rensen FJ, KruhÃ¸ffer M, Laurberg S, Kauppinen S, Ã˜rntoft TF, Andersen CL. Diagnostic and prognostic microRNAs in stage II colon cancer. Cancer Res. 2008 Aug 1;68(15):6416-24.
10. Chen X, Guo X, Zhang H, Xiang Y, Chen J, Yin Y, Cai X, Wang K, Wang G, Ba Y, Zhu L, Wang J, Yang R, Zhang Y, Ren Z, Zen K, Zhang J, Zhang CY. Role of miR-143 targeting KRAS in colorectal tumorigenesis Oncogene. 2009 Mar 12;28(10):1385-92. Epub 2009 Jan 12.
11. Motoyama K, Inoue H, Takatsuno Y, Tanaka F, Mimori K, Uetake H, Sugihara K, Mori M. Over- and under-expressed microRNAs in human colorectal cancer. Int J Oncol. 2009 Apr;34(4):1069-75.
12. Bandres E, Agirre X, Bitarte N, Ramirez N, Zarate R, Roman-Gomez J, Prosper F, Garcia-Foncillas J. Epigenetic regulation of microRNA expression in colorectal cancer. Int J Cancer. 2009 Dec 1;125(11):2737-43.
13. Baffa R, Fassan M, Volinia S, O'Hara B, Liu CG, Palazzo JP, Gardiman M, Rugge M, Gomella LG, Croce CM, Rosenberg A. MicroRNA expression profiling of human metastatic cancers identifies cancer gene targets. J Pathol. 2009 Oct;219(2):214-21.

14. Huang ZM, Yang J, Shen XY, Zhang XY, Meng FS, Xu JT, Zhang BF, Gao HJ. MicroRNA expression profile in non-cancerous colonic tissue associated with lymph node metastasis of colon cancer. J Dig Dis. 2009 Aug;10(3):188-94.
15. Diosdado B, van de Wiel MA, Terhaar Sive Droste JS, Mongera S, Postma C, Meijerink WJ, Carvalho B, Meijer GA. MiR-17-92 cluster is associated with 13q gain and c-myc expression during colorectal adenoma to adenocarcinoma progression. Br J Cancer. 2009 Aug 18;101(4):707-14.
16. Arndt GM, Dossey L, Cullen LM, Lai A, Druker R, Eisbacher M, Zhang C, Tran N, Fan H, Retzlaff K, Bittner A, Raponi M. Characterization of global microRNA expression reveals oncogenic potential of miR-145 in metastatic colorectal cancer. BMC Cancer. 2009 Oct 20;9:374.
17. Huang Z, Huang D, Ni S, Peng Z, Sheng W, Du X. Plasma microRNAs are promising novel biomarkers for early detection of colorectal cancer. Int J Cancer. 2010 Jul 1;127(1):118-26.
18. Sarver AL, French AJ, Borralho PM, Thayanithy V, Oberg AL, Silverstein KA, Morlan BW, Riska SM, Boardman LA, Cunningham JM, Subramanian S, Wang L, Smyrk TC, Rodrigues CM, Thibodeau SN, Steer CJ. Human colon cancer profiles show differential microRNA expression depending on mismatch repair status and are characteristic of undifferentiated proliferative states. BMC Cancer. 2009 Nov 18;9:401.
19. Navon R, Wang H, Steinfeld I, Tsalenko A, Ben-Dor A, Yakhini Z. Novel rank-based statistical methods reveal microRNAs with differential expression in multiple cancer types. PLoS One. 2009 Nov 25;4(11):e8003.
20. Ahmed FE, Jeffries CD, Vos PW, Flake G, Nuovo GJ, Sinar DR, Naziri W, Marcuard SP. Diagnostic microRNA markers for screening sporadic human colon cancer and active ulcerative colitis in stool and tissue. Cancer Genomics Proteomics. 2009 Sep-Oct;6(5):281-95.
21. Akao Y, Nakagawa Y, Hirata I, Iio A, Itoh T, Kojima K, Nakashima R, Kitade Y, Naoe T. Role of anti-oncomirs miR-143 and -145 in human colorectal tumors. Cancer Gene Ther. 2010 Jun;17(6):398-408. Epub 2010 Jan 22.
22. Wang YX, Zhang XY, Zhang BF, Yang CQ, Chen XM, Gao HJ. Initial study of microRNA expression profiles of colonic cancer without lymph node metastasis. J Dig Dis. 2010 Feb;11(1):50-4.
23. Earle JS, Luthra R, Romans A, Abraham R, Ensor J, Yao H, Hamilton SR. Association of microRNA expression with microsatellite instability status in colorectal adenocarcinoma. J Mol Diagn. 2010 Jul;12(4):433-40. Epub 2010 Apr 22.
24. Chen Y, Song Y, Wang Z, Yue Z, Xu H, Xing C, Liu Z. Altered expression of MiR-148a and MiR-152 in gastrointestinal cancers and its clinical significance. J Gastrointest Surg. 2010 Jul;14(7):1170-9. Epub 2010 Apr 27.
25. Lagerstedt KK, Kristiansson E, LÃ¶nnroth C, Andersson M, IresjÃ¶ BM, Gustafsson A, Hansson E, Kressner U, Nordgren S, Enlund F, Lundholm K. Genes with relevance for early to late progression of colon carcinoma based on combined genomic and transcriptomic information from the same patients. Cancer Inform. 2010 Apr 23;9:79-91.
26. Link A, Balaguer F, Shen Y, Nagasaka T, Lozano JJ, Boland CR, Goel A. Fecal MicroRNAs as novel biomarkers for colon cancer screening. Cancer Epidemiol Biomarkers Prev. 2010 Jul;19(7):1766-74. Epub 2010 Jun 15.
27. Kulda V, Pesta M, Topolcan O, Liska V, Treska V, Sutnar A, Rupert K, Ludvikova M, Babuska V, Holubec L Jr, Cerny R. Relevance of miR-21 and

miR-143 expression in tissue samples of colorectal carcinoma and its liver metastases. Cancer Genet Cytogenet. 2010 Jul 15;200(2):154-60.
28. Li XM, Wang AM, Zhang J, Yi H. Down-regulation of miR-126 expression in colorectal cancer and its clinical significance. Med Oncol. 2011 Dec;28(4):1054-7. Epub 2010 Jul 31.
29. Liu L, Chen L, Xu Y, Li R, Du X. microRNA-195 promotes apoptosis and suppresses tumorigenicity of human colorectal cancer cells. Biochem Biophys Res Commun. 2010 Sep 17;400(2):236-40. Epub 2010 Aug 19.
30. Koga Y, Yasunaga M, Takahashi A, Kuroda J, Moriya Y, Akasu T, Fujita S, Yamamoto S, Baba H, Matsumura Y. MicroRNA expression profiling of exfoliated colonocytes isolated from feces for colorectal cancer screening. Cancer Prev Res (Phila). 2010 Nov;3(11):1435-42. Epub 2010 Oct 19.
31. Chiang Y, Song Y, Wang Z, Chen Y, Yue Z, Xu H, Xing C, Liu Z. Aberrant expression of miR-203 and its clinical significance in gastric and colorectal cancers. J Gastrointest Surg. 2011 Jan;15(1):63-70. Epub 2010 Nov 10.
32. Slattery ML, Wolff E, Hoffman MD, Pellatt DF, Milash B, Wolff RK. MicroRNAs and colon and rectal cancer: differential expression by tumor location and subtype. Genes Chromosomes Cancer. 2011 Mar;50(3):196-206. doi: 10.1002/gcc.20844. Epub 2010 Dec 16.
33. Cheng H, Zhang L, Cogdell DE, Zheng H, Schetter AJ, Nykter M, Harris CC, Chen K, Hamilton SR, Zhang W. Circulating plasma MiR-141 is a novel biomarker for metastatic colon cancer and predicts poor prognosis. PLoS One. 2011 Mar 17;6(3):e17745.
34. AkÃ§akaya P, Ekelund S, Kolosenko I, Caramuta S, Ozata DM, Xie H, Lindforss U, Olivecrona H, Lui WO. miR-185 and miR-133b deregulation is associated with overall survival and metastasis in colorectal cancer. Int J Oncol. 2011 Aug;39(2):311-8. doi: 10.3892/ijo.2011.1043. Epub 2011 May 13.
35. Kahlert C, Klupp F, Brand K, Lasitschka F, Diederichs S, Kirchberg J, Rahbari N, Dutta S, Bork U, Fritzmann J, Reissfelder C, Koch M, Weitz J. Invasion front-specific expression and prognostic significance of microRNA in colorectal liver metastases. Cancer Sci. 2011 Oct;102(10):1799-807. Epub 2011 Aug 4.
36. Chang KH, Miller N, Kheirelseid EA, Lemetre C, Ball GR, Smith MJ, Regan M, McAnena OJ, Kerin MJ. MicroRNA signature analysis in colorectal cancer: identification of expression profiles in stage II tumors associated with aggressive disease. Int J Colorectal Dis. 2011 Nov;26(11):1415-22. Epub 2011 Jul 8.
37. Balaguer F, Moreira L, Lozano JJ, Link A, Ramirez G, Shen Y, Cuatrecasas M, Arnold M, Meltzer SJ, Syngal S, Stoffel E, Jover R, Llor X, Castells A, Boland CR, Gironella M, Goel A. Colorectal cancers with microsatellite instability display unique miRNA profiles. Clin Cancer Res. 2011 Oct 1;17(19):6239-49. Epub 2011 Aug 15.
38. Kalimutho M, Del Vecchio Blanco G, Di Cecilia S, Sileri P, Cretella M, Pallone F, Federici G, Bernardini S. Differential expression of miR-144* as a novel fecal-based diagnostic marker for colorectal cancer. J Gastroenterol. 2011 Dec;46(12):1391-402. Epub 2011 Aug 24.
39. Mosakhani N, Sarhadi VK, Borze I, Karjalainen-Lindsberg ML, SundstrÃ¶m J, RistamÃ¤ki R, Osterlund P, Knuutila S. MicroRNA profiling differentiates colorectal cancer according to KRAS status. Genes Chromosomes Cancer. 2012 Jan;51(1):1-9. doi: 10.1002/gcc.20925. Epub 2011 Sep 15.
40. Bartley AN, Yao H, Barkoh BA, Ivan C, Mishra BM, Rashid A, Calin GA, Luthra R, Hamilton SR. Complex patterns of altered MicroRNA expression during the

adenoma-adenocarcinoma sequence for microsatellite-stable colorectal cancer. Clin Cancer Res. 2011 Dec 1;17(23):7283-93. Epub 2011 Sep 23.

41. Wang LG, Gu J. Serum microRNA-29a is a promising novel marker for early detection of colorectal liver metastasis. Cancer Epidemiol. 2012 Feb;36(1):e61-7. Epub 2011 Oct 21.
42. Vickers MM, Bar J, Gorn-Hondermann I, Yarom N, Daneshmand M, Hanson JE, Addison CL, Asmis TR, Jonker DJ, Maroun J, Lorimer IA, Goss GD, Dimitroulakos J. Stage-dependent differential expression of microRNAs in colorectal cancer: potential role as markers of metastatic disease. Clin Exp Metastasis. 2012 Feb;29(2):123-32. doi: 10.1007/s10585-011-9435-3. Epub 2011 Nov 26.

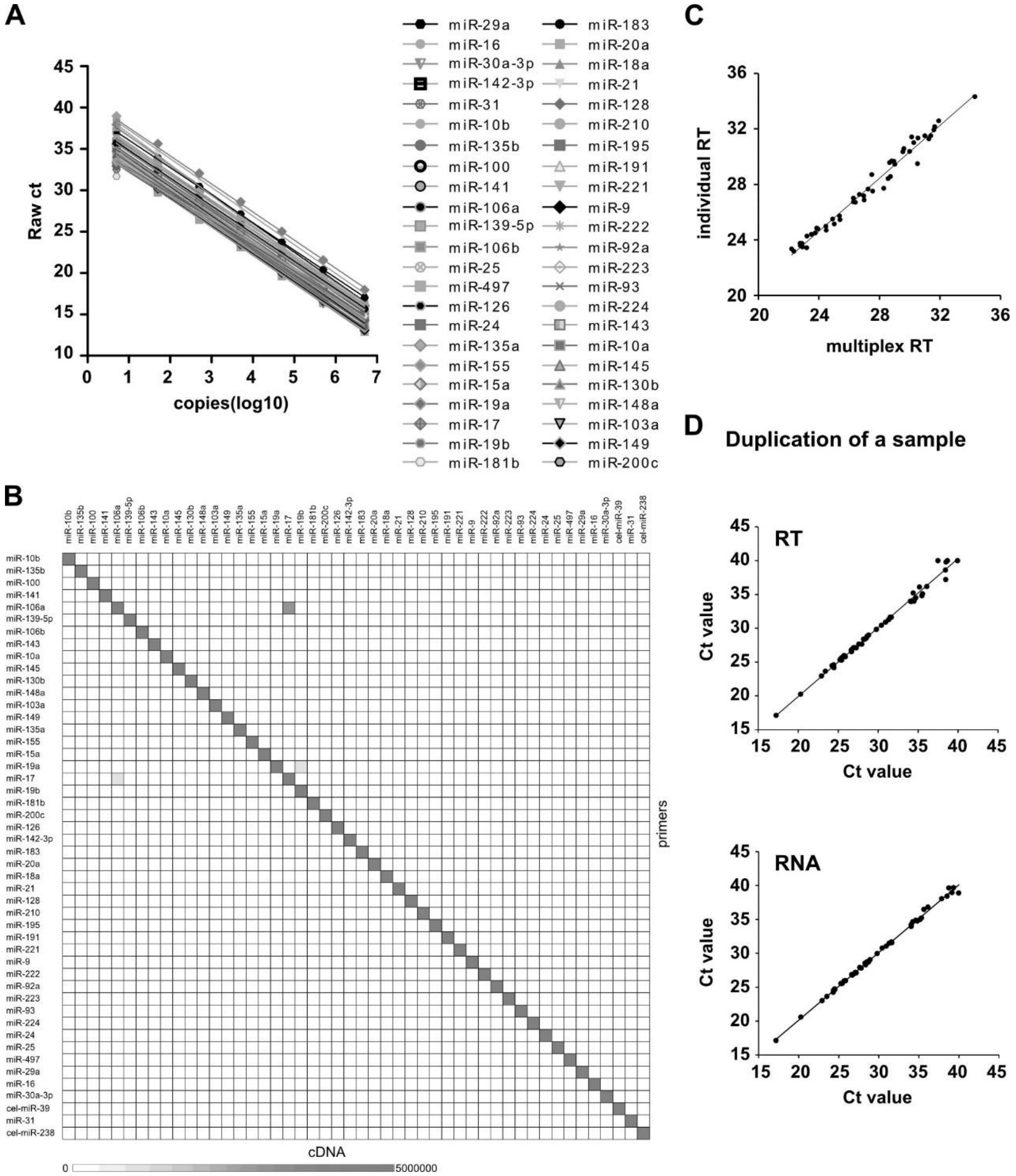

Supplementary Figure 2. Analytic performance of stem- [illegible] RT-qPCRs for quantitation of 46 miRNAs. **A**. Linearity of individual miRNA qPCR ranges from 5 to $5x10^6$ copies. **B.** Specificity check by performing individual miRNA PCR with $5x10^6$ copies of 46 synthetic cDNA. The color of square cell depends on the corresponding miRNA copy number. **C.** Efficiency of reverse-transcription by 46 single-plex or multiplex reaction. Ct value of two types of reaction shows similar results. **D.** Precision check by performing multiplex stem-loop RT-qPCRs with duplication at RT step and RNA extraction step using one plasma sample. Comparison of 46 miRNA Ct values of duplicated experiments show very high correlation.

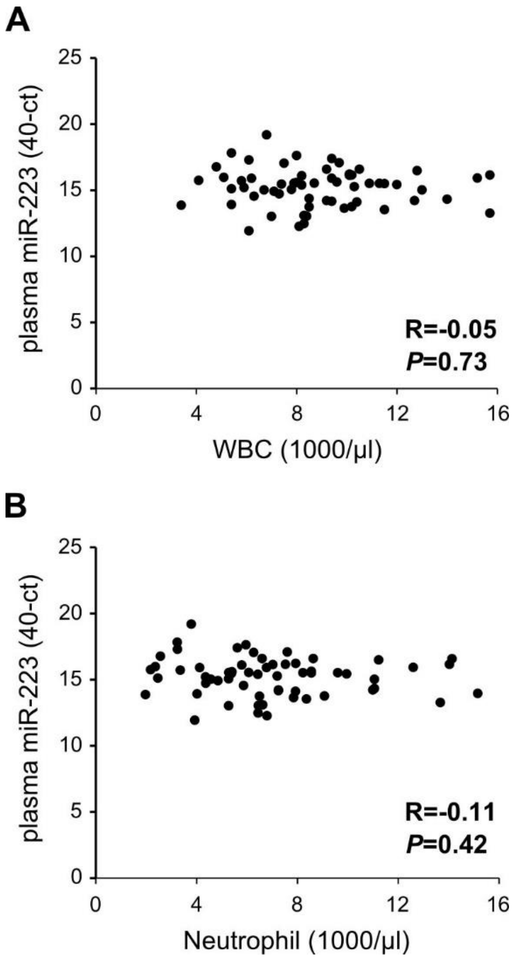

Supplementary Figure 3. Spearman's correlation (R) between the level of plasma miR-223 and WBC (A) and Neutrophil count (B) in 62 CRC patients. No significant correlation between them was observed.

Supplementary Table 2. Spearman's correlation between the levels of differentially expressed miRNAs in stool and plasma in total set and the age of healthy control and CRC group. No significant correlation between miRNA expression and age.

		Healthy control		CRC	
	miRNA	Correlation Coefficient	*p*-value	Correlation Coefficient	*p*-value
stool	miR-106b	-0.068	0.559	0.030	0.639
	miR-92a	0.033	0.775	-0.013	0.835
	miR-223	-0.045	0.701	-0.004	0.947
	miR-16	-0.070	0.546	0.070	0.272
plasma	miR-18a	-0.100	0.274	0.015	0.855
	miR-92a	-0.044	0.632	-0.063	0.442
	miR-221	-0.057	0.535	-0.110	0.175
	miR-223	-0.078	0.395	-0.143	0.079
	miR-191	-0.144	0.115	-0.159	0.050
	miR-24	-0.078	0.394	-0.134	0.099
